# Protein Networks Reveal Detection Bias and Species Consistency When Analysed by Information-Theoretic Methods

**DOI:** 10.1371/journal.pone.0012083

**Published:** 2010-08-18

**Authors:** Luis P. Fernandes, Alessia Annibale, Jens Kleinjung, Anthony C. C. Coolen, Franca Fraternali

**Affiliations:** 1 Randall Division of Cell and Molecular Biophysics, King's College London, London, United Kingdom; 2 Department of Mathematics, King's College London, London, United Kingdom; 3 Division of Mathematical Biology, MRC National Institute for Medical Research, London, United Kingdom; Keio University, Japan

## Abstract

We apply our recently developed information-theoretic measures for the characterisation and comparison of protein–protein interaction networks. These measures are used to quantify topological network features *via* macroscopic statistical properties. Network differences are assessed based on these macroscopic properties as opposed to microscopic overlap, homology information or motif occurrences. We present the results of a large–scale analysis of protein–protein interaction networks. Precise null models are used in our analyses, allowing for reliable interpretation of the results. By quantifying the methodological biases of the experimental data, we can define an information threshold above which networks may be deemed to comprise consistent macroscopic topological properties, despite their small microscopic overlaps. Based on this rationale, data from yeast–two–hybrid methods are sufficiently consistent to allow for intra–species comparisons (between different experiments) and inter–species comparisons, while data from affinity–purification mass–spectrometry methods show large differences even within intra–species comparisons.

## Introduction

Comparative genomics has revolutionised the study of biology by shifting its focus from component characterisation to the study of systemic properties. We envisage that in the near future the comparison of interactomes of different species might drive a similar, if not even more powerful transformation [1], [2]. Interaction maps (otherwise here named interactomes, referring to the entire set of molecular interactions in the cell) can reveal important mechanistic principles that may guide further progress in the understanding of cellular function, and of dysfunction leading to disease [3]–[5]. One would expect that the availability of interactome maps for several organisms could give new insights into how biological diversity is embedded in the networks' functionality [6]. In contrast to genomic data, however, the available interactome data are still far from complete and of limited reproducibility [7], [8]. One can compare protein-protein interaction network (PPIN) datasets by simply counting the fraction of common interactions, referred to as ‘overlap’. However, the overlap values found are typically small, which prohibits a meaningful comparison [9]. Alternative approaches have therefore been proposed. Some focus on identifying conserved ‘modules’ or recurrent geometrically defined motifs, envisaged to capture biological and functional properties of the underlying networks [10], [11] or common functional cores of ancestral origin [12], [13]. Others employ alignment strategies where phylogenetic information is derived by the identification of paralogues [14], [15]. These studies illustrate the additional information provided by comparative interactomics, beyond comparative genomics, and the benefit of intra-species comparison [16].

However, to progress further in comparative interactomics, a serious problem needs to be resolved. Recent analyses of PPINs sparked a debate about the influence of the experimental method on the quality and biological relevance of the interaction data [17]. Current experimental techniques, such as yeast two-hybrid (Y2H) and co–affinity purification combined with mass spectrometry (AP–MS), sample subsets of the interaction data space [18]. These subsets show very limited overlap [7]. Moreover, AP–MS interaction data are non-binary by nature for any multi-component complex; their conversion to binary pair-interactions is non–trivial and relies on processing protocols that may introduce further biases in the final screening output [17], [19]. It is vital that we understand to what extent observed discrepancies between different networks reflect sampling biases of their experimental methods, as opposed to topological features due to biological functionality.

In information-theoretic terms overlap is not a good measure of the similarity between two sampled networks, just as the size in bits of a file does not give its true information content. It is therefore natural to explore the potential of information-theoretic measures for comparing interaction networks. These require a systematic characterisation of network topologies, which is a general prerequisite in network science [20], and formulations in terms of network sample probabilities, based on macroscopic topological features. One is thus led to study the relationship between structured random graph ensembles and real biological signalling networks. The rationale is that PPIN data should be regarded as noisy samples of a true underlying network, and that the family of such samples is best described and studied statistically as a structured random graph ensemble with controlled macroscopic topological features. If the control parameters of the random graph ensemble can be derived from sufficiently accurate and complete network data, it is in principle possible to calculate (asymptotically) explicit formulae for entropies and complexities, and for information-theoretic distances between network families.

In recent years there have been efforts to define and generate random graphs whose topological features can be controlled and tailored to experimentally observed networks. In Perez-Vicente and Coolen [21] a parameterised random graph ensemble was defined where graphs have not only a prescribed degree distribution but also prescribed degree correlations. We have recently been able to show [22] that this graph ensemble (described in [21]) can be tailored asymptotically to the generation of graphs with any prescribed degree distribution and any prescribed degree correlation function. Moreover, for this ensemble the combinatorial problem of calculating the network complexities and information-theoretic distances between network families can be solved analytically. The result is a novel, practical and precise mathematical framework, that allows for the large-scale analysis and unbiased comparison of PPINs from different species and measured with different techniques. Here we apply this formalism to an extensive range of PPINs, and show that it provides a quantitative window on interactome data. The topological network distance is applied here to cluster network data and to estimate intra-species similarity for differently detected interaction data and inter-species distances within and between experimental methods. In particular, the presence of methodological data biases and the topological similarity between networks with small microscopic overlap can be detected clearly and at low computational cost.

## Results

### The PPIN data taken from literature

**Figure 1:**
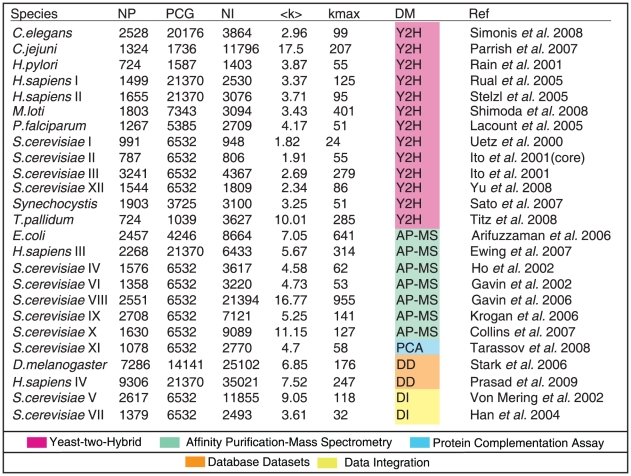
Table of all 25 experimental PPIN datasets analysed in this study, corresponding to 11 different species. These included nine eukaryotic organisms (*Caenorhabditis elegans*, *Drosophila melanogaster*, *Homo sapiens*, *Plasmodium falciparum* and *Saccharomyces cerevisiae*), and six bacterial species (*Campylobacter jejuni*, *Escherichia coli*, *Helicobacter pylori*, *Mesorhizobium loti*, *Synechocystis* and *Treponema pallidum*). Abbreviations stand for: NP, Number of Proteins; NI, Number of Interactions; PCG, Number of Protein Coding Genes; AD, Average Degree; 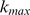), Maximum Degree; Ref, References. Most datasets were derived from high–throughput experiments detected by either Y2H [29], [46]–[55] or AP-MS [56]–[62]; we also included a recent PCA dataset [63]. In addition we analysed a series of consolidated datasets that include both high-throughput experiments and literature-mined small-scale studies [64], [65]. The Ito et al. (2001) [47] dataset was divided into two sets: a high confidence set defined as the ‘core’ set and a low confidence set, as suggested by the authors. The Collins et al. (2007) [58] dataset consists of the raw purifications identified by the Krogan et al. (2006) [59] and Gavin et al. (2002) [56] studies, but re-analysed by a different scoring and clustering algorithm. Lastly, for completeness we have also included two commonly used yeast datasets: the Dong et al. (2004) [66] network, which is a consolidated dataset referred to in the literature as the ‘Filtered Yeast Interactome’ (comprising experimentally determined and *in silico* predicted interactions), and the von Mering et al. (2002) [67] dataset, which has been assembled from two catalogues of yeast protein complexes (the MIPS catalogue and the Yeast Protein Database catalogue).

In the table of Fig. 1 we give a comprehensive table of all the PPIN data that are used in the analysis presented in this paper, colour coded according to the experimental method that was used. The table lists for each PPIN dataset various simple quantitative characteristics, such as the number of nodes (NP), interactions (NI), protein coding genes (PCG) and the average (AD) and maximum (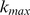) degree 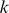, which is defined as the number of interaction partners of a node.

### Macroscopic characterisation: degree statistics and degree–degree correlations

We characterise each PPIN by its degree distribution 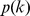 and its normalised degree–degree correlations (DDCs) 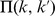 (the latter quantity gives the likelihood that two proteins with degrees 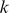 and 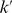 interact, relative to what would be found in random networks with the same degree distribution but uncorrelated degrees). The precise definitions are given in the Methods section. A value 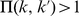 indicates that protein pairs with degrees 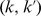 interact more than what one would expect on the basis of their degree values, whereas if 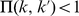 they interact less than expected; either case would signal topological information beyond that encoded in the degree statistics alone. We applied a weak Gaussian smoothening to these functions, to prevent probabilities from being strictly zero. The resulting numerical differences in the macroscopic quantities are irrelevant for the presented data. Various quantities have been proposed in the past for characterising the structure of networks. One reason for choosing the macroscopic features 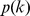 and 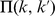 is that many of the previously proposed quantities are either similar or equivalent to (or expressible in terms of) 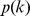 and 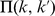. Examples are degree sequences [23], degree distributions [24], degree correlations [25], and assortativity [26]. Some authors, however, used measures that are qualitatively different, such as clustering coefficients [27] and so-called community structures [28].

Before embarking upon an information-theoretic analysis of our PPIN datasets, based on the macroscopic topological features captured by 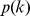 and 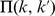, we first verify that for these datasets the function 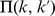 actually contains topological information, *i.e.* deviates significantly from the value one. It would also be useful to know how these topological features may have evolved; one would expect that closely related species should also have PPINs with more similar topological features.

**Figure 2:**
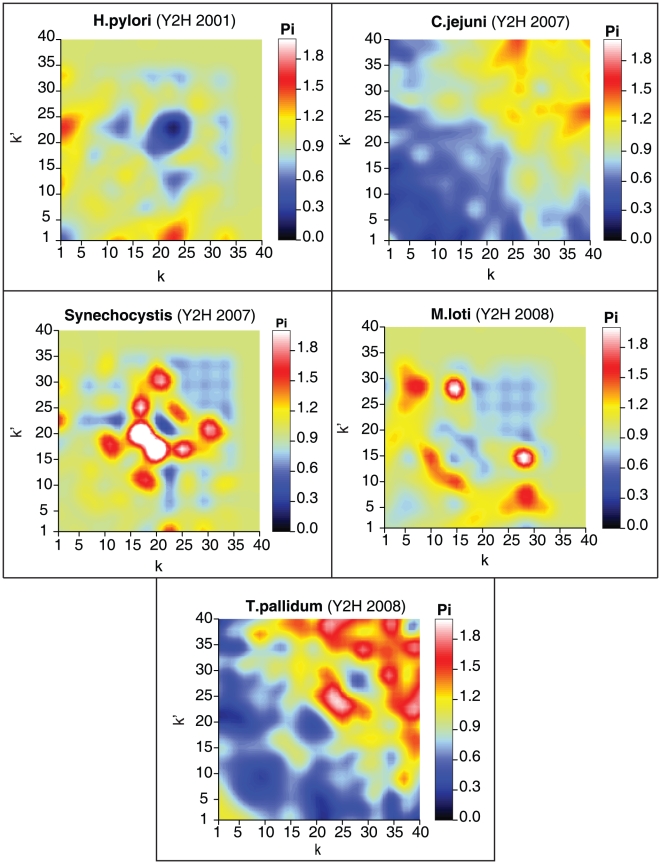
Heat map representations of the normalised DDC function 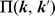 of bacterial PPINs. For each combination of 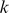 and 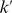, 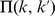 gives the likelihood that two proteins with degrees 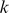 and 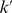 interact, relative to what would be found in appropriate null models (random networks with the same degree distribution but uncorrelated degrees). The degree axes 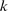 and 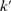 were truncated to the value 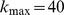. White regions indicate strongly enhanced propensity for protein-protein interaction (values of 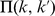 larger than expected on the basis of degree information alone) while dark regions indicate reduced protein-protein interaction (values of 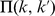 smaller than expected on the basis of the degree distribution alone).

In Fig. 2 we show the normalised DDCs for the bacterial species in our dataset collection in heat map representation, with a colour scale ranging from black (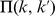 close to zero) to white (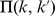 very large). Since 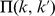 is a symmetric function, the plots are always symmetric around the main diagonal. The figure reveals that generally the normalised DDCs deviate significantly from those of random networks with the same degree statistics, where one would have found 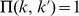 throughout (modulo small fluctuations). Apparently there is significant topological information contained in the degree correlations, and this is seen to give rise to quite diverse patterns for the different bacterial species. Some species (*e.g. Synechocystis*) appear to exhibit normalised DDCs mostly higher than the random level, some (*e.g. C. jejuni*) exhibit normalised DDCs that are mostly lower, whereas for *e.g. T. pallidum* one observes strong deviations from the random level in either direction. The most closely related bacterial species in our datasets are *H. pylori* and *C. jejuni*, which both belong to the *Campylobacterales* genus, yet this is not reflected in their DDC patterns. On the contrary, the *H. pylori* network exhibits only minor DDC deviations from the random level, unlike *C. jejuni*. Similarly, comparison of the networks of *C. jejuni*, *T. pallidum* and *H. pylori*, which all belong to the *Proteobacteria* phylum family (comprising the majority of gram-negative bacteria), does not reveal any conserved pattern.

**Figure 3:**
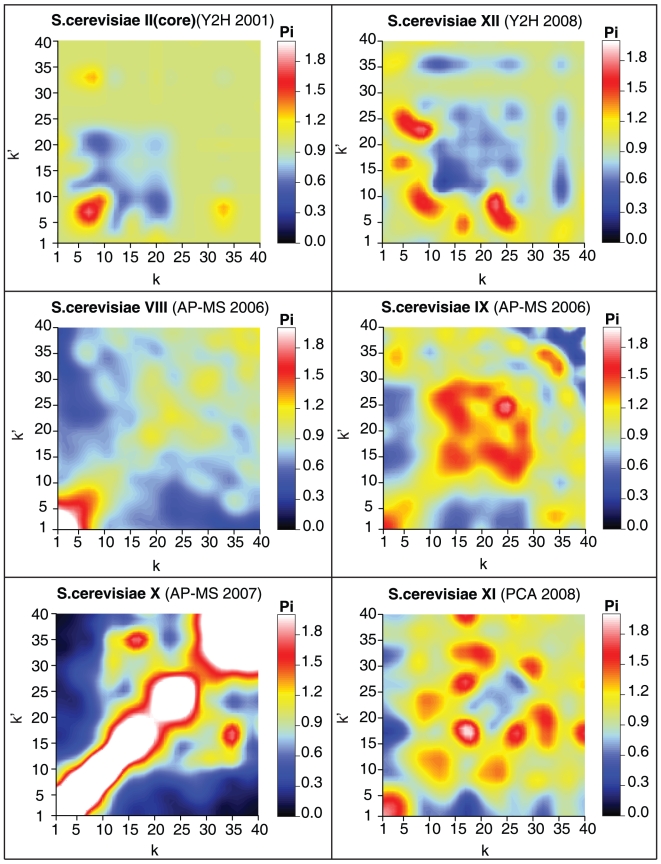
Heat map representations of the normalised DDC function 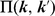 of yeast PPINs. Definitions and conventions are identical to those of Fig. 2.

Even more strikingly and worryingly, consistent DDC fingerprints are not even observed for plots that refer to datasets of the same species. In Fig. 3 we show the normalised DDCs for yeast, which has been the focus of most of the large-scale PPIN determinations so far. The plots in Fig. 3, displayed in order of experimental determination and date, do not suggest conservation of the macroscopic topological PPIN features.

A hint at a possible explanation emerges if one compares only plots that refer to the same experimental technique. The DDC patterns then appear more similar, differing mostly in terms of the strengths of the deviations from the random level, which increase roughly with the time of publication of the PPIN dataset. Compare for example *S. cerevisiae* II (core) to *S. cerevisiae* XII (both obtained *via* Y2H), and *S. cerevisiae* VIII to *S. cerevisiae* X (both obtained *via* AP–MS). The interactions reported on the *S. cerevisiae* X dataset were in fact derived from the raw purification data of two AP-MS datasets (*S. cerevisiae* VIII and *S. cerevisiae* IX), but these data were processed using a different scoring and clustering protocol. A strong positive correlation is observed along the diagonal for this latter dataset, indicating an enhancement of interactions between nodes of similar degree. In general, the AP-MS datasets show stronger DDC patterns than the Y2H datasets (this we also observed for the *H. sapiens* datasets) although the regions where the main deviations from the random level occur are quite different.

### Assortativity

The assortativity of a network is a quantity that measures in a single number the extent to which the degrees of connected nodes are correlated with each other; it can be expressed in terms of 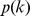 and 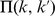 *via* a simple formula.

Positive assortativity indicates positive correlation between the degrees of connected nodes (implying that nodes prefer to interact with other nodes of similar degree) while the contrary is true for negative assortativity (here high degree nodes prefer to interact with low degree nodes). We can therefore view and use the assortativity as a single parameter that summarises part of the full information provided in our DDC plots. To assess the relevance of any observed topological feature in a network, it must be compared to its frequency of observation in appropriate null models. These are benchmark networks, generated randomly and with uniform probabilities from the set of *all* networks that share specified features with the network under study. In this paper we choose as null models random networks that share with our biological PPINs the degree distribution 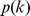. Many properties of these null models can be calculated analytically if the number of nodes is sufficiently large; for instance, lacking further topological structure, our null models would have 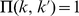 for all 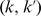, and zero assortativity. In this section we compare our observations for each dataset to null models that have been generated *via* numerical simulations (by careful re-shuffling of the network under study; see the Methods section for a detailed description of the randomisation algorithm), to capture also finite size effects.

**Figure 4:**
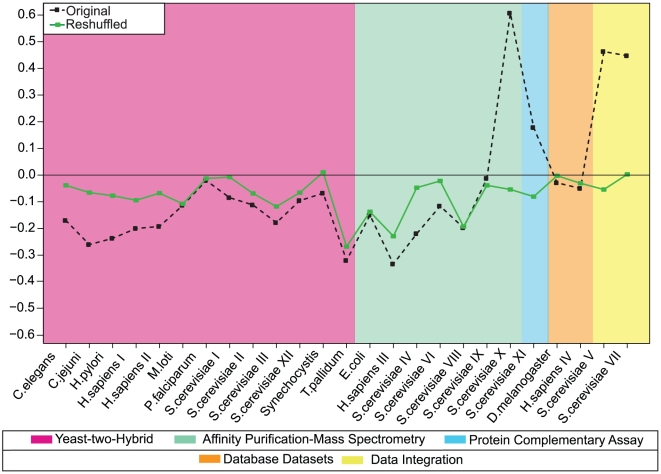
Assortativities as calculated for both the biological PPINs (black line) and their randomised versions (or ‘null models’, green line). The randomised networks have degree distributions identical to their biological counterparts, but are otherwise fully random (the randomisation seeks to remove any DDCs initially present). A positive assortativity implies that nodes prefer to interact with other nodes of similar degree, whereas a negative assortativity implies that high degree nodes prefer to interact with low degree nodes.

In Fig. 4 we plot in black the assortativities of our PPIN datasets (Original), together with those of their randomisations (null models) in green (Reshuffled). Most sets are seen to have slightly negative assortativity values, indicating a weak preference for interactions between nodes with different degrees. The main deviants from this trend are *S. cerevisiae* X, *S. cerevisiae* V and *S. cerevisiae* VII, with strong positive assortativity. This is consistent with Fig. 3, where the *S. cerevisiae* X dataset is indeed distinguished by the presence of consistently high values of 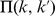 along the main diagonal, signalling a strong preference for interactions between nodes with similar degrees. The assortativities of the null models (in green) are expected to be closer to zero than those of the real PPINs. This is indeed true for most cases, although for some networks (*e.g. M. loti*, *P. falciparum*, *E. coli*, and *S. cerevisiae* VIII) the assortativity differences between the original networks and their null models are negligible. In sufficiently large networks, all correctly generated null models would exhibit zero assortativity, so any deviation of the green line from zero in Fig. 4 must reflect finite size effects or effects caused by slow relaxation (see ‘Definition and generation of null models’ in the Methods) during the randomisation process (or both). In Fig. 4 the deviations are most likely due to finite size effects; this can be concluded upon measuring the Hamming distances between the original networks and their null models (which measure the extent of microscopic dissimilarity between the two, see Fig. S1), which show no evidence for insufficient relaxation in the null model generation.

### Degree complexity and wiring complexity

**Figure 5:**
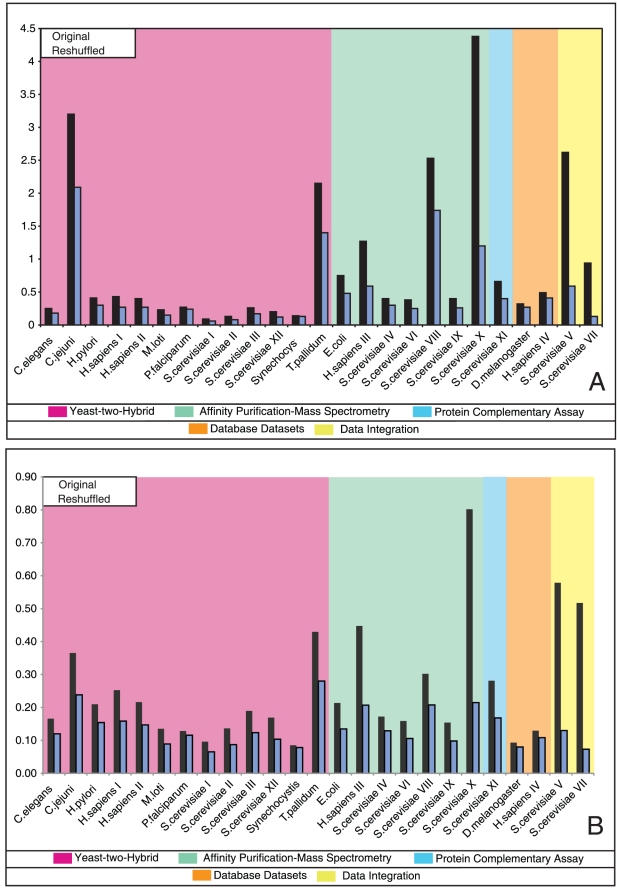
Wiring complexities as calculated for both the biological PPINs (black bars) and their randomised versions (or ‘null models’, grey bars). The randomised networks have degree distributions identical to their biological counterparts, but are otherwise fully random. The wiring complexity measures the topological information contained in a network's normalised DDC function 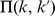, beyond that in its degree statistics 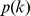. (A) The network complexity is computed per network node. The average degree of each network contributes to its complexity value. (B) The network complexity is computed per link, which removes the dependency on the average degree and reduces complexity value (note the different ordinate scales (A) and (B)).

We now turn to information-theoretic quantifiers of PPIN structure, applying the methods developed in Annibale et al. [22]. One of these is the network complexity, which (modulo finite size effects) measures the amount of topological information contained in a network's degree statistics and DDCs. It consists of two contributions, both of which can be expressed explicitly in terms of the functions 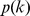 and 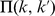. The first is the degree complexity, measuring the information revealed by knowledge of 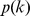 alone. The second is the wiring complexity, measuring the information revealed by subsequent knowledge of 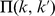. See the Methods section for precise definitions. In Fig. 5A we plot the wiring complexities, as black bars, for our experimental datasets (Original), together with those of their randomisations (null models) in grey (Reshuffled). In panel (a) the network complexity is computed ‘per node’ as given in equation 2 (Methods) and it takes into account the average degree of the network, while the complexity ‘per link’ in panel (b) is independent of the average degree. For our dataset it appears more appropriate to use the ‘per link’ complexity, because the relative differences between Y2H and AP–MS*S. cerevisiae* networks are smaller. The AP–MS networks tend to have higher wiring complexities than the Y2H ones, although less so in the ‘per link’ plot, except for *C. jeuni* and *T. pallidum*. The latter, however, are special in that around 80% of their predicted encoded proteins have at least one assigned interaction (this percentage can be seen as an approximation of the ‘coverage’ of the network), which is the highest value among all datasets studied; this may explain differences between these two networks and other Y2H datasets. Similarly to what was observed for assortativities, *S. cerevisiae* X is again seen to stand out with an extremely high wiring complexity, consistent with the strong degree correlations observed earlier in Fig. 3.

### Information-theoretic clustering

**Figure 6:**
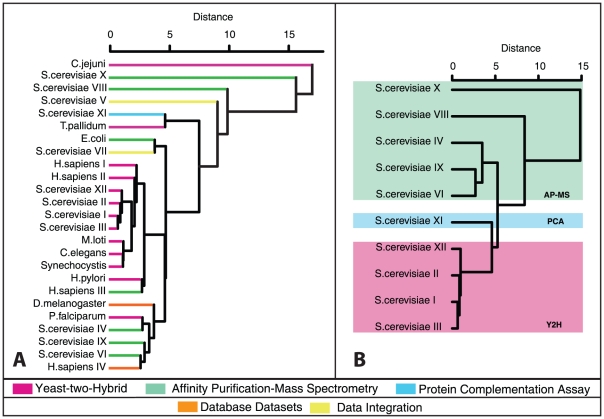
Network comparison by clustering using the full information-theoretic distance measure of **equation 9** (see Methods) [**22**]. This distance is expressed explicitly in terms of the degree statistics and normalised DDC functions of the networks concerned. Panel A: Dendrogram calculated for an extensive collection of PPINs, covering a wide range of species. Panel B: Dendrogram for PPINs of the same species, *viz. S. cerevisiae*. Both trees were constructed using our proposed distance metric in a hierarchical clustering routine (see Methods). In both panels we decorated the clusters using the same colour scheme used throughout this paper to indicate the different experimental detection techniques (see bottom legend of the figure). The data integration datasets (*S. cerevisiae* V and *S. cerevisiae* VII) were excluded from panel b, since they are composed of interactions detected by a variety of different techniques.

A second information-theoretic tool derived in Annibale et al. [22] is a transparent formula for an information-theoretic ‘distance’ between any two networks, once more expressed explicitly in terms of the functions 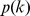 and 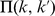 of the networks concerned. This network distance is the symmetrised Kullback-Leibler divergence of the maximum entropy graph distributions with degree distributions and degree correlations identical to those of the two networks. We can use this mutual distance measure to cluster our PPIN datasets, and construct dendrograms analogous to phylogenetic trees. In Fig. 6A we show the resulting information-theoretic dendrogram for the full collection of all our PPIN data sets. The pariwise distance matrices of the AP-MS and Y2H data sets are provided in Tables S1 and S2, respectively. In Fig. 6B we limit our analysis to single-technique *S. cerevisiae* data sets only (excluding *S. cerevisiae* V and *S. cerevisiae* VII, which are the result of integrating datasets detected by a variety of different techniques). The results of these analyses are quite revealing. Those data sets which were most strongly criticised in the past for having worryingly small overlaps [29], for example the Y2H data sets *S. cerevisiae* I *versus* II and *H. sapiens* I *versus* II, are now unambiguously found to be topologically similar after all.

We also observe that the full collection of PPINs group primarily by detection method, so at least for the presently available PPIN datasets, any biological similarities (whether or not based on evolutionary relationship) are overshadowed by methodological biases. This is particularly evident in the central subgroup (central pink leaves) in Fig. 6A, which clusters almost exclusively Y2H datasets and comprises a wide range of species. The methodological biases are also obvious in the intra-species comparison of *S. cerevisiae* depicted in Fig. 6B. The largest sub–group distance within this *S. cerevisiae* tree is the one between two AP–MS datasets that have been post–processed differently (the top two within the green box). Also, the single PCA network is separated from the AP–MS and Y2H subgroups. We can now summarise the two, in our view, most important observations:

- PPINs of the same species and measured *via* the same experimental method are statistically similar, and more similar than networks measured *via* the same method but for different species. Apparently, the former exhibit similar *macroscopic* topological features, despite the small microscopic overlap of the individual PPINs. The information-theoretic network distance is therefore a useful macroscopic descriptor of similarity.
- PPINs measured *via* the same experimental method cluster together, revealing a bias introduced by the methods that is seen to overrule species-specific information. Although methodological biases have been acknowledged in the literature, we are now in a position to quantify their impact: the bias is proportional to the excess distances between the *S. cerevisiae* networks measured by AP-MS compared to those measured by Y2H (Fig. 6B).

Therefore, a species tree based on data from two different experimental methods yields an inconsistent picture (see Fig. 6A), in which the wanted contributions of the ‘species distance’ are modulated with the unwanted contributions of the ‘methodological distance’ originating from sampling biases.

**Figure 7:**
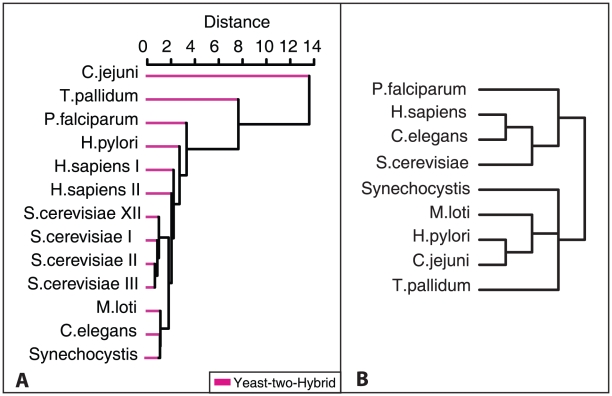
Network comparison by clustering. A: Y2H network cluster using the information-theoretic distance measure. B: Reference species tree provided by the NCBI taxonomy common tree service.

A clearer picture is obtained when trees based on data from a single experimental method are constructed. Since the Y2H data appear less biased than the AP-MS data, a multi-species tree of Y2H networks is shown in Fig. 7A juxtaposed to a reference tree (Fig. 7B). Comparison of the trees reveals that the network distance measure correctly assigns short distances between *H. sapiens*, *C. elegans* and *S. cerevisiae*, but misassigns short distances between these species and *M. loti* or *Synechocystis*. Therefore one may deduce that the ‘biological signal’ captured by the network complexity difference is indeed strong enough for some of the networks in our data set to place them correctly on the tree, while others would require more data completeness to reach a comparable signal. For example, the two correctly placed bacteria *C. jejuni* and *T. pallidum* show a relatively high complexity (Fig. 5B).

Finally, in order to separate the contributions of the degree distribution and the DDCs to the distance information in generating the dendrograms shown in Fig. 6, we have also performed the same computations on the basis of a simplified information-theoretic distance measure for PPINs, which would have been the result of characterising all PPINs by their degree distributions alone. The result is shown in Fig. S2.

### Network size effects

**Figure 8:**
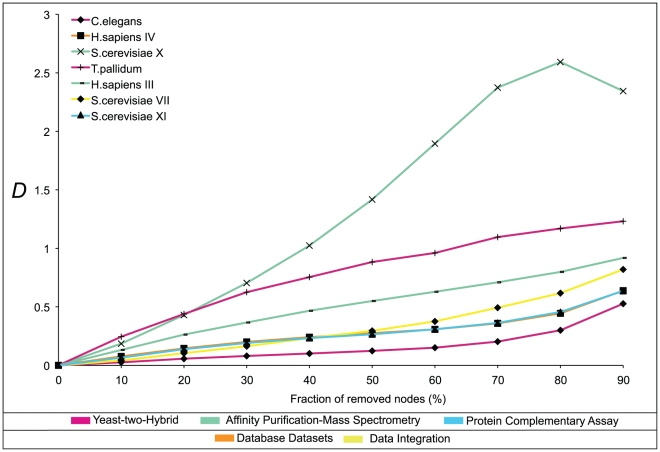
The effect of random sub-sampling on the network topology. Networks were modified by random removal of nodes to various degrees, given as fraction of nodes in the original network in the range from 10 to 90% in 10% increments. The information-theoretic distance between the original network and the sub-sampled network is plotted over the degree of node removal. A selection of typical curves is shown. Networks with high complexity show a large distance to the original network.

The mathematical framework operates on statistical grounds and the precision of the results depends on the sample size. We have already pointed out finite size effects in the assortativity of some reshuffled networks. To assess the robustness of the macroscopic network properties *versus* the variation of the sampled data and the sample size, we have performed a sub-sampling experiment. Each network was modified by randomly removing a certain fraction of its nodes (from 10 to 90% in 10% increments) and the distance between the modified (sub-sampled) and the original network was plotted as a function of the degree of node removal (Fig. 8). The plot shows an exemplary collection of networks. The sensitivity of the networks towards the sample size is correlated with their complexity. Sub-sampled networks with high complexity yield larger distances to their original versions than those with low complexity. This is not surprising, as the distance is a measure of the complexity difference. However, the particular curve shapes in Fig. 8 are related to the distribution of node links. For example, the extreme behaviour of *S. cerevisiae* X is owing to its large number of hub-hub links (see Fig. 3), while the *S. cerevisiae* XI network with a flatter DDC distribution is relatively robust to node removal.

The effects of sub-sampling, random and non-random, on the network statistics have been explored by others [8], [30], but without consideration of DDCs. The curves in Fig. 8 provide an error estimate for the sub–sampling effects on the information-theoretic properties discussed in this paper. This regards the DDC plots in Figs. 2,3, the network complexities in Fig. 5 and the trees in Figs. 6,7.

## Discussion

In this paper we investigated the potential of recently introduced mathematical framework for quantifying and comparing the topologies of PPINs by systematic application to publicly available PPIN datasets. This framework provides exact and explicit measures of network complexity and information-theoretic distances between any two networks. In addition, in benchmarking empirical measurements on PPINs we used null models generated *via* recently developed rigorously unbiased algorithms.

Our methods involve a macroscopic characterisation of PPINs by their degree statistics and DDCs. Degree correlation properties have been used in the past to highlight topological features of networks and to suggest general principles governing functional mechanisms in the interactome [31]–[35]. These particular studies focused on regularities in interactions between high *versus* low degree nodes; unfortunately they did not agree on the nature and interpretation of such regularities, in particular on the role of high–degree (hub) proteins. Maslov and Sneppen (2002) [31] argued that suppression of hub–hub interactions is a ‘universal feature’ of robust molecular networks, that reflects compartmentalisation and modularity, characteristics of cellular processes. In contrast, Batada et al. (2006) [32] and Ivanic et al. (2008) [35] did not observe hub–hub interaction suppression, but suggested instead that hub–hub interactions play an important role in the underlying biological processes. An intermediate position was taken by Friedel and Zimmer (2007) [33], who generated artificial versions of biological networks and argued that neither type of degree–weighted behaviour is favoured.

We find that degree-degree correlations provide important and consistent information on PPIN topologies, but it is crucial that they are normalised correctly and that one uses robust and systematic methods for extracting this information. Normalisation of DDCs is usually based on comparison against appropriate randomised networks (null models). The unbiased generation of such null models, however, is nontrivial. Popular randomisation protocols such as ‘edge-swapping’ are now known to carry the risk of biased sampling, see [36]. The reason why we avoided the inconsistencies of previous studies [31]–[35] appears to be that, rather than normalising DDCs *via* numerical randomisation, we use an exact mathematical formula for the DDCs of large unbiased random graphs. Our normalised DDCs are by definition unbiased, and not subject to numerical normalisation noise. Where we employ null models for reasons other than normalisation, we use exact algorithms for generating unbiased null models that have only recently become available. Under these improved conditions one does detect reproducible DDC patterns, with an overall preference for high–low degree interactions. However, the variation of DDC patterns, even within the same species and detection method, precludes general conclusions about their origin in the underlying biological mechanisms. This type of inference would require improved (in terms of completeness and error rate) interaction data for several related networks.

The first information-theoretic tool we applied to the PPIN datasets was the formula for a network's complexity recently derived in Annibale et al. [22]. It has two contributions: a term representing the complexity embedded in the degree statistics (the degree complexity), and a second term representing the complexity embedded in the DDCs (the wiring complexity). The wiring complexity quantifies the extent to which DDCs are prominent in a network, similar to the assortativity measuring the nature of the lowest order correlations (if present). The two quantities provide complementary information. One can easily imagine higher order DDCs in PPINs (*e.g.* nonlinear relationships between the degrees of preferred protein partners) that could not be picked up by the assortativity but would still be detected by the wiring complexity. In fact this is already visible in the presently analysed PPIN data. Comparison of Fig. 4 to Fig. 5A shows that, while those datasets with nontrivial assortativities also have high wiring complexities, there are several further PPINs with a high wiring complexity but only a relatively modest assortativity.

The second information-theoretic tool we applied was a formula for an information-theoretic distance between networks. Like the complexity, the formula is expressed explicitly in terms of the networks' degree statistics and DDCs, and, based on macroscopic statistical features, it avoids the problems with the more primitive overlap-based network dissimilarity measures. Application of this second tool to our datasets resulted in a pairwise distance table, which we used to cluster the PPINs. The results, summarised in a dendrogram, are very revealing. Those data sets which were most strongly criticised in the past for having small overlaps, for example the Y2H data sets of *S. cerevisiae*, are now unambiguously found to be topologically similar. Furthermore, our method shows clearly that the PPINs group primarily by detection method, so biological similarities based on evolutionary relationship are presently overshadowed by methodological biases. These biases have been the centre of an active debate in recent years; the problems which they generate and methods to overcome these have been described recently, *e.g.* [9], [17], [37], [38].

In particular, an often overlooked aspect of data derived from AP–MS experiments is the influence of the post-processing protocol on the final binary interaction map. This crucial aspect is now starting to be addressed by different groups [39]–[41], and we expect more accurate data to emerge in the near future. Our information-theoretic tools are thus very timely: they provide the required resolution and precision in the assessment and comparison of new PPIN data, and in evaluating the progress of the experimental methods. Being able to quantify biases accurately is a prerequisite for their systematic removal.

One should keep in mind that biological systems are not necessarily perfect, and that the presence in PPINs of non-selected, non-functional PPIs is to be expected [42]; the interpretation of interactome data will therefore always have to take account of noise. This again suggests that information-theoretic methods, with their rigorous probabilistic basis, should be seen as the appropriate tools in PPIN analysis. One could also envisage these methods being used to guide experimental efforts aimed at remedying the present under-sampling of PPINs, by predicting on statistical grounds the properties of missing network nodes and interactions.

In conclusion, we believe to have succeeded in

- supplying the biological and bio-informatics communities with a new generation of precise and user-friendly computational tools with which to quantify PPIN topologies and test new protocols for the removal of experimental biases from PPIN datasets, and
- demonstrating by a systematic application of these tools to publicly available datasets that the present protein network data are strongly biased by their experimental methods, while still exhibiting species–specific similarity and reproducibility.

We hope and anticipate that in the near future the accuracy and sensitivity of experiments will improve substantially, alongside a further sharpening of the mathematical and computational tools for their analysis, allowing for meaningful comparisons of interactomes.

## Materials and Methods

The following section gives a complete reference of the formulae used in this study. The central equations 2, 7 and 9 have been published in a recent work by the authors [22] in the context of parametrised random graph ensembles. They are repeated here in commented form to aid the reader.

### Mathematical definitions

#### Degree distribution

Given a protein-protein interaction network with 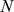 nodes, we label its proteins by Roman indices 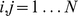, and represent the microscopic interaction information as a symmetric matrix 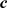 with entries 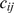, where 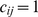 if 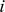 interacts with 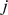, and 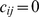 otherwise (with 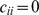 for all 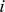). The degree of a node 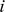 is then defined as 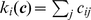, and the degree distribution of the PPIN is defined as
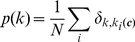
(1)(here 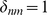 if 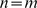 and 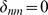 if 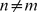).

#### Degree–degree correlation (DDC)

The average degree in the PPIN is given by 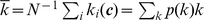. The normalised DDC function 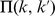 of the network is defined as the ratio between the probability that two randomly picked nodes in 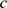 with degrees 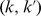 are found to be connected, divided by what this probability would have been in large *random* networks with the same degree distribution as 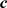. The probabilities for large random networks can be calculated analytically, see *e.g.* [43]. This results in the following definition:
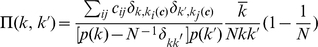
(2)This quantity was plotted for our PPIN datasets in heat-map form in Figs. 2 and 3. Any statistically significant deviation from 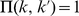 signals the presence of non-trivial DDCs. Both 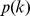 and 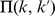 are macroscopic quantities that can be measured directly and at low computation cost.

#### Assortativity

The assortativity 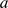 (as plotted in Fig. 4 for our PPIN datasets) is defined [26] as the magnitude of the normalised correlations for the joint probability 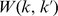 of finding a randomly drawn interaction in the graph 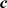 connecting nodes with degrees 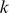 and 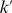 respectively, *viz.*

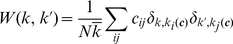
(3)Upon defining averages over this measure as 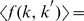 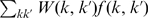, and using the symmetry of 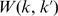 as well as the relation 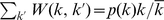, one has
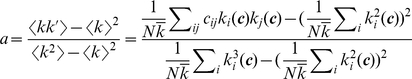
(4)The relation between 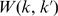 and 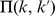 is
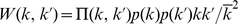
(5)which is why the assortativity can be written as a function of 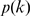 and 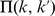:
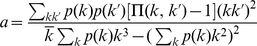
(6)


#### Hamming distance

The Hamming distance is defined as 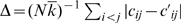, where the binary interaction variables 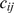 define the original PPIN and the variables 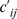 represent its randomisation.

### Information-theoretic tools

#### Degree complexity and wiring complexity

Using methods from random graph theory and statistical mechanics the following explicit formula was derived for the information-theoretic complexity 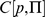 per node of non-directed networks (such as PPINs) with degree distribution 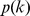 and normalised degree-degree correlations 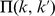:
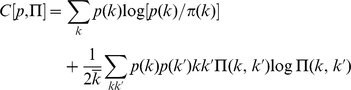
(7)where 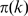 is the Poissonian distribution with average degree 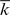:
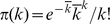
(8)The first term in (7) is called the degree complexity; the second term, which would be zero in our null models, is called the wiring complexity. This latter quantity was plotted in Fig. 5A.

#### Network distance

Similar calculations led also to an explicit formula for an information-theoretic distance 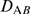 between any two non–directed networks A and B (such as PPINs), characterised by the structure functions 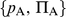 and 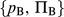, respectively:
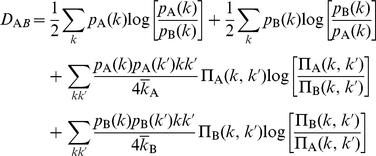
(9)with 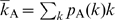 and 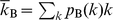. The distance (9) was used to calculate the dendrogram of Fig. 6. A simplified distance that no longer takes DDC information into account is obtained upon removing the last two lines from (9), leaving an expression that involves only the two degree distributions 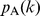 and 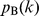; this simplified distance definition was used to calculate the dendrogram of Fig. 7.

### Definition and generation of null models

Given an observed 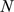-node PPIN with degree distribution 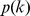, we define its associated null model as a graph drawn randomly and with uniform probabilities from the set of *all* 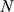-node graphs with degree distribution 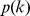, so the probability of any graph 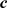 being generated as null model for the PPIN under study must be
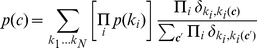
(10)


The issue of sampling uniformly the desired space of graphs is non–trivial. Naive application to the original PPIN of the popular method of ‘edge-swapping’ (or ‘graph shuffling’) would indeed upon equilibration produce randomised graphs, but these might not be sampled uniformly; biased sampling would invalidate any inference based on comparing observations in real PPINs to those in randomised graphs. In this paper we used the general and exact Markov chain Monte Carlo (MCMC) method for generating random graphs proposed in Coolen et al. [36], which is based on edge–swaps [44] but involves nontrivial move acceptance probabilities. Most graph randomisation protocols, including the one used in this paper, are defined *via* a degree–preserving MCMC dynamics in the space of graphs, which is defined such that it produces a relaxation towards an equilibrium state where all acceptable graphs are generated with prescribed probabilities. This dynamics must be run for a sufficient duration of time to guarantee that all transients in the MCMC have died down and the desired equilibrium state has indeed been reached. In this paper we have used equilibration times such that the number of accepted transitions in the MCMC exceeded 100 per link, which (upon systematic monitoring of a number of key observables in the graphs) was found to be adequate to ensure equilibration of the Markov chain.

### Numerical practicalities

After measuring a graph's degree distribution 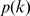 and normalised DDC functions 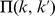 we applied to both functions a weak Gaussian smoothening, resulting in the new functions
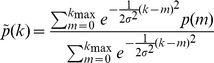
(11)

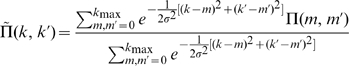
(12)with diffusion width 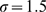. The reason for doing this is that it prevents probabilities from being strictly zero, which (while reflecting only finite size effects) would cause problems in the distance measure (9). A further benefit of this smoothening is that it removes some of the finite size noise from the images in Figs. 2 and 3.

Dendrograms, as shown in Fig. 5A and Fig. 6, were computed using information-theoretic network distances (9) in the hierarchical clustering routine ‘hclust’ of the R environment [45] with the ‘average’ agglomeration method.

Finally, in plotting the normalised degree-degree correlations of PPINs in Figs. 2 and 3, we chose to limit ourselves to 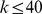. The reason is that while proteins with larger degrees certainly exist in the networks studied, the limited number of these no longer justify the interpretation of quantities such as 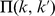 as clean estimators of (normalised) probabilities; this would require more data points in the large 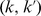 regions.

## Supporting Information

Figure S1 This figure shows for each PPIN the normalised Hamming distance between the original network and its null model. The null models were obtained for each PPIN by application of the exact Markov Chain Monte Carlo randomisation protocol of Coolen et al. 2009. The Hamming distance Δ is defined in such a way that it equals zero if the two networks are strictly identical, and equals one if the two networks are statistically independent (apart from the values of their degrees, which are preserved by the randomisation). The effect of insufficient equilibration of the randomisation protocol would be marked by Hamming distances significantly less than one. This figure supports our confidence that the equilibration time which we used in the randomisation algorithm, being 100 accepted moves per protein interaction, were adequate.
(0.45 MB EPS)

Figure S2 Network comparison by clustering using a simplified information-theoretic distance without the contribution of DDCs. Definitions and conventions are identical to those in Figure 6.
(0.46 MB EPS)

Table S1 Triangular matrix of information theoretic network distance between the AP-MS datasets.
(0.00 MB TXT)

Table S2 Triangular matrix of information theoretic network distance between the Y2H datasets.
(0.00 MB TXT)
